# Prolonged Physical Inactivity in Older Adult Couples: A Dyadic Analysis Using Actigraphy

**DOI:** 10.1093/geroni/igaa066

**Published:** 2020-12-30

**Authors:** Chao-Yi Wu, Lyndsey M Miller, Rachel N Wall, Zachary T Beattie, Lisa C Silbert, Jeffrey A Kaye

**Affiliations:** 1 Department of Neurology, Oregon Health & Science University, Portland, USA; 2 Oregon Center for Aging & Technology (ORCATECH), Oregon Health & Science University, Portland, USA; 3 School of Nursing, Oregon Health & Science University, Portland, USA; 4 Department of Neurology, Veterans Affairs Portland Health Care System, Portland, Oregon, USA

**Keywords:** Exercise/Physical activity, Independence, Technology

## Abstract

**Background and Objectives:**

Many older adults remain inactive despite the known positive health implications of physical activity (improved mood, reduced mortality risk). Physical inactivity is an interdependent phenomenon in couples, but most research examines physical inactivity at the individual level. We estimated the average amount of prolonged physical inactivity for older adult couples and, using dyadic analysis, identified physical and mental health determinants thereof.

**Research Design and Methods:**

Forty-six heterosexual older adult couples (age = 70.61 ± 6.56) from the Veterans Integrated Service Network 20 cohort of the Collaborative Aging Research using Technology (CART) initiative were included. The average number per day of prolonged inactive periods (no step counts or sleep activity for ≥30 min) was estimated using actigraphy data collected over a month.

**Results:**

Multilevel modeling revealed that, *within* couples, there was no significant difference between partners in the average amount of inactive periods (*p* = .28). On average *across* couples, males and females had an average of 6.90 ± 2.02 and 6.56 ± 1.93 inactive periods per day, respectively. For males, older age was the only variable associated with more inactive periods (β = 0.15, *p* = .002). For females, having more depressive symptoms in both dyad members was associated with fewer inactive periods (female: β = −0.30, *p* = .03; male: β = −0.41, *p* < .001), and more dependence in completing their own instrumental activities of daily living predicted more inactive periods (β = 2.58, *p* < .001).

**Discussion and Implications:**

Viewing couples’ activity as an interdependent phenomenon, rather than individual, provides a novel approach to identifying pathways to reduce inactivity in older adults, especially when focusing on the mental health and level of independence within the couple.

**Translational Significance:** Reducing physical inactivity is a growing interest in the aging population when exercise becomes a challenging option. Viewing couples’ activity as a unit is a promising approach to yield lifestyle changes and promote healthy practices. This unique study and high-resolution data offered the opportunity to explore the dyadic influence of physical inactivity within real-world home settings for a month. The consideration of demographics (age), mental health issues (depression), and instrumental activity of daily living dependence within and between dyads can be built upon current interventions to better develop tailored interventions for older adults.

## Background and Objectives

Reducing physical inactivity is one of the most important health practices in which older adults can engage to support a positive mood, strengthen the immune system, and reduce morbidity (i.e., chronic illness burden) and mortality risk (Lynch et al., 2010). Although the health benefits of physical activity are well-established, approximately 25.4% to 35.3% of older adults are insufficiently physically active (Watson et al., 2016). It has been estimated that older adults spend 5.3–9.4 hr of awake time per day inactive, which was reported to diminish the health benefits of light-intensity physical activities (Harvey et al., 2015). The high prevalence and negative health implications of physical inactivity highlight the importance of interventions to reduce the amount of inactivity in the lifestyles of an aging population.

In general, the health behavior of one member of a couple is associated with the health behavior of the other member (Cornelius et al., 2016). Engaging in physical activity has been found to be an interdependent phenomenon in couples (Pauly et al., 2020; Pettee et al., 2006) and is negatively affected by caregiving for one’s partner during times of illness (Queen et al., 2019). Yet, the physical *inactivity of couples* has rarely been studied, especially among older adults. Increasing physical activity and decreasing physical inactivity are distinct health-promoting behaviors (Pauly et al., 2020), and they can coexist to influence one’s health over time. While older adults perceive poor health as the leading barrier to physical activity or exercise (e.g., pain, frailty, injuries; Schutzer & Graves, 2004), redirecting the focus on decreasing physical inactivity or sedentariness may be more feasible and applicable for some older adults looking to adopt a healthier lifestyle.

In order to inform pathways to reduce inactivity, it is important to identify the determinants of physical inactivity at the couple level. This is critical to understanding the behavioral interdependence of two related individuals living in the same household. The determinants of physical inactivity at the individual level have previously been found to include higher body mass index (Cornelius et al., 2016), more comorbidities (Vancampfort et al., 2017), lower self-efficacy (Maher & Conroy, 2016), less motivation (Rollo et al., 2016), and social isolation (Tully et al., 2019). Yet, studies have rarely examined determinants of physical inactivity at the couple level.

Further, the interrelatedness of couples’ physical inactivity, chronic conditions, and emotional well-being remains largely unstudied in the scientific literature. There is strong theoretical support for taking a dyadic perspective to chronic illness and physical activity in later life, as demonstrated in the Developmental Contextual Model of Couples Coping with Chronic Illness (Berg & Upchurch, 2007). The model points to the sociocultural factors (e.g., gender), proximal contextual factors (e.g., chronic conditions, marital quality), and developmental stage (e.g., age) as influential in older adult couples’ coping and adjustment. Because couples are exposed jointly to each other’s stressful events (e.g., chronic conditions), dyadic coping and the resulting lifestyle changes (e.g., physical inactivity) are also likely to happen jointly. It is important to consider that the physical and emotional well-being of one member of the couple may affect the physical inactivity of the other member of the couple (cross-partner effect) (Monin et al., 2016). For example, functional limitations in instrumental activities of daily living (IADLs) due to the chronic condition of one member of the couple might lead to more sedentariness or more hands-on care (less sedentariness) of the other member, while emotional distress (e.g., depression, anxiety) of one member may either decrease or increase physical inactivity on the other member, depending on the way in which the couple is adjusting their lifestyle.

Thus, the aim of the study was to estimate the average amount of physical inactivity for older adult couples continuously over a month and to identify individual and cross-partner determinants thereof. A month of data would provide more valid and reliable patterns of typical physical inactivity rhythms than the conventional 1–2 weeks of data, which may be insufficient (Berger et al., 2008). We hypothesized that there would be cross-partner effects of chronic conditions, dependence in IADLs, and depressive symptoms on physical inactivity in older adult couples. By expanding the determinants of physical inactivity at the couple level, one can better understand health-relevant outcomes and gain insight into the potential interpersonal pathways within couples to make better everyday health choices.

## Research Design and Methods

The current study is a secondary analysis of older adult couples who were originally recruited from the Veterans Integrated Service Network 20 (Northwest VISN 20) in the Pacific Northwest to participate in the Collaborative Aging Research using Technology (CART) initiative (Kaye et al., 2018). Study approval was obtained from the Institutional Review Board at Oregon Health & Science University and the VA Portland Health Care System (IRB 00017123). The CART initiative began enrolling participants in this longitudinal study in 2017 and is ongoing as of 2020.

### Inclusion/Exclusion Criteria

In order to be eligible in the CART VA cohort, one member of each household needed to be a Veteran of the United States Military, aged over 57 years old, living in a residence larger than a one-room apartment, living either alone or with a spouse/partner, and without dementia (age and education adjusted Montreal Cognitive Assessment [MoCA] > 18 [Nasreddine et al., 2005] and Clinical Dementia Rating [CDR] scale 0 or 0.5). Households needed to have a reliable broadband internet connection. Exclusion criteria included more than two people living in the participant’s residence and a condition that would limit their physical participation at the entry to the study (e.g., wheelchair-bound). The exclusion criteria of participants who lived in residences with more than one other person was specified to explore the interdependence and dyadic relationship of spouses’ or partners’ data without adding other coresidential family members (thereby increasing other sources of variance). Spouses or partners who lived in the same household were required to consent to the study. Informed consent and interviews were conducted for members of dyads separately. All participants agreed to wear the actigraphy devices for the duration of the study period. In addition to the homes with couples enrolled in CART, there were also homes with single older adult participants in the parent study. These participants were excluded from the current analysis due to the nature of the dyadic research question.

### Outcome Measure

#### Physical inactivity

Commercial Withings actigraphy watches were used to estimate physical inactive time (Tudor-Locke et al., 2013). Consecutive zero step counts detected from the actigraphy were used as a measure of inactivity. An inactive period was defined as no step counts or sleep activity for ≥30 min, following a previous study methodology (van Dommelen et al., 2016). Inactive periods were used instead of inactive time because studies have suggested prolonged inactive periods (>30 min) are indicative of a higher risk for all-cause mortality (Diaz et al., 2017). Various criteria have been used to classify nonwear time in actigraphy (Gibbs et al., 2015). To account for the variability of nonwear time across participants, we examined the distribution of the minutes of prolonged inactive periods per individual. Inactive periods that exceeded 3 *SD*s of the mean duration of inactive periods were treated as nonwear time and were excluded from the analysis. Because the CART initiative is an ongoing longitudinal observational study that was still enrolling at the time of the analysis, we used baseline data for the current study. The first-month data may be influenced by the awareness of device monitoring; therefore, we extracted the second-month data for the analysis. The rate of wearing compliance was calculated by the number of days with step counts divided by the total days of the month.

#### Independent variables

All independent variables were measured at baseline, prior to the measurement of activity data. Participants with missing data were excluded from the analysis.

Depressive symptoms were measured by the Geriatric Depression Scale (GDS; Yesavage et al., 1982). The GDS has 15 items; each item was scored as YES (1) or NO (0). The total score ranges from 0 to 15 with a higher score indicating more severe depressive symptoms. The GDS has a high degree of internal consistency (alpha coefficient = 0.94) and reliability (reliability coefficient = 0.94; test–retest reliability = 0.85; Yesavage et al., 1982).

Anxiety symptoms were measured by the Generalized Anxiety Disorder 7 (GAD-7; Spitzer et al., 2006). The GAD-7 has 7 items, each item was scored from Not at all (0) to Nearly every day (3). The total score ranges from 0 to 21, with a higher score indicating more severe anxiety symptoms. The GAD-7 has excellent internal consistency (Cronbach α = 0.92) and good test–retest reliability (intraclass correlation = 0.83; Spitzer et al., 2006).

Cognitive function was measured by the MoCA (Nasreddine et al., 2005). The MoCA assesses cognitive domains, including short-term memory recall, visuospatial abilities, executive function, attention, concentration, working memory, language, and orientation. The MoCA has 30 items, with a sum score ranging from 0 to 30. A higher score indicates better cognitive function. The MoCA has excellent sensitivity (90%) and specificity (87%) to detect mild cognitive impairment (Nasreddine et al., 2005).

Functional independence was measured by the Older Americans Resources and Services (OARS) Instrumental Activities of Daily Living (IADL) Scale (Fillenbaum & Smyer, 1981). The OARS-IADL Scale is widely used with older adults and has 6 items related to functional independence in common household activities (telephone use, shopping, meal preparation, housework, medication management, and financial management); each item was rated on a 3-point Likert-like scale, from 0 “Without help” to 2 “Completely unable to.” The total score ranges from 0 to 12, with a higher score indicating greater dependency in IADL. Interrater reliability (0.67–0.87) and validity (0.60–0.83) have been demonstrated for the OARS (Fillenbaum & Smyer, 1981).

Illness and comorbidity were measured by the modified Cumulative Illness Rating Scale-Geriatric (CIRS-G; Miller et al., 1992). The modified CIRS-G has 14 items; each item was rated on a 5-point Likert-like scale from 1 “None” to 5 “Extremely severe.” The total score ranges from 14 to 70, with a higher score indicating more severe comorbidity. The CIRS-G has good interrater reliability (intraclass correlations = 0.78–0.88; Miller et al., 1992).

Gait speed was measured by a complete 15-foot out and back gait test. The instruction was to walk at your usual pace. The total time (seconds) to complete the 15-foot walk was recorded, with more time indicating a slower gait speed.

### Data Analysis

Dyadic multilevel modeling was performed using the software program Hierarchical Linear Modeling, version 7. The multivariate outcomes model for dyadic data was used to simultaneously model both dyad members’ physical inactivity, while controlling for the dependency (shared variances) in the dyadic data (Barnett et al., 1993). The unconditional models estimated the average within-dyad values (fixed effects) and the variability around the averages (random effects) for both males’ and females’ average daily number of physically inactive periods. The conditional models included independent variables to help explain the variability around the averages. Parameter values were estimated using full-information maximum likelihood, which has the advantage of handling any missing data. However, in this sample, there were no missing data in the overall outcome of average daily periods of physical inactivity (see measures section for the handling of data missing at the daily/item level).

#### Unconditional model

Variation in the average amount of physical inactivity at the within-dyad level was modeled in the equation,


$$\begin{array}{l} \left[INACT_{ij}=\beta_{1j}MALE_{ij}+\beta_{2j}FEMALE_{ij}+r_{ij}\right] \end{array}$$

where INACT_*ij*_ represents the outcome score *i* in dyad *j.* MALE is an indicator variable taking on a value of 1 if the response was obtained from the male in the couple or taking on a value of 0 if the response was obtained from the female in the couple. Similarly, FEMALE is an indicator variable taking on a value of 1 if the response was obtained from the female in the couple or taking on a value of 0 if the response was obtained from the male in the couple. The latent true scores of ratings of physical inactivity for males and females are represented by β _1*j*_ and β _2*j*_, respectively. The within-dyad residuals, *r*_*ij*_, are estimated separately for males and females and represent the variance around the intercepts for males and females.

#### Conditional model

Variation at the between-dyad level was modeled in the equations,


$$\begin{array}{ll} \beta_{1j}= & \;\;\gamma_{10}+\gamma_{11}DEP1_{j}+\gamma_{12}DEP2_{j}+\gamma_{13}IADL1_{j}\\ & +\gamma_{14}IADL2_{j}+\gamma_{15}AGE_{j}+u_{1j} \end{array}$$

$$\begin{array}{ll} \beta_{2j}= & \;\;\gamma_{20}+\gamma_{21}DEP1_{j}+\gamma_{22}DEP2_{j}+\gamma_{23}IADL1_{j}\\ & +\gamma_{24}IADL2_{j}+\gamma_{25}AGE_{j}+u_{2j} \end{array}$$

where the parameters for latent true scores of males and females are the outcome variables, and independent variables (DEP1 & DEP2 = male & female depressive symptoms; IADL1 & IADL2 = male and female IADLs) are introduced to explain the variance in the outcomes (males’ and females’ average physical inactivity). Potential determinants were identified by a Spearman correlation *r* > 0.25 (at least weak to moderate correlation; Steiner et al., 2010). If a health variable (e.g., depressive symptoms) was correlated with inactive periods in one gender, both genders’ health variables were included in the analysis to allow for examination of cross-partner effects. Because both dyad members’ depressive symptoms were associated with physically inactive periods in females, we also explored the possibility of a within-dyad interaction of men and women’s depressive symptoms (DEP1 * DEP2 = interaction term entered into conditional models) on physical inactivity periods in older women.

## Results

A total of 68 households were eligible and consented to the CART initiative VA cohort. Single-person dwelling households (*n* = 21) and one female with missing data (*n* = 1) were excluded and in total 46 couples were included in the current analysis. All couples enrolled happened to be heterosexual. On average, participants were 70.6 (*SD* = 6.6) years in age and had 14.4 (*SD* = 2.2) years of education. All the couples had at least one chronic condition reported. Additional participant characteristics are presented in Table 1. Supplementary Table 1 in Online Supplementary Material includes bivariate correlations between dependent and independent variables.

**Table 1.** Demographic Characteristics

	Male *n* = 46 (100%)	Female *n* = 46 (100%)
Characteristics	Mean ± *SD* or # of participants (%)	
Age	73.11 ± 6.37	68.42 ± 5.88
Years of education	14.46 ± 2.21	14.26 ± 2.17
Rurality		
Urban	14 (30.4%)	
Large rural	15 (32.6%)	
Rural	17 (37.0%)	
Depressive symptoms, GDS (range 0–15)^a^	1.87 ± 2.26	1.43 ± 1.92
Anxiety symptoms, GAD-7 (range 0–21)^a^	2.91 ± 4.25	1.63 ± 2.59
IADL independence, OARS (range 0–12)^a^		
0	40 (87.0%)	39 (84.8%)
1	3 (6.5%)	7 (15.2%)
≥2	3 (6.5%)	0 (0.0%)
Comorbidity, CIRS-G (range 14–70)^a^	23.65 ± 3.73	21.26 ± 3.19
Cognitive function, MoCA (range 0–30)	22.73 ± 3.36	24.23 ± 3.30
Wearable data (days)	26.16 ± 6.06	26.17 ± 6.07
Wearable compliance rate (%)	0.87 ± 0.20	0.87 ± 0.20
Percentage of nonwear bouts (%)^a^	0.02 ± 0.10	0.02 ± 0.01

*Notes*: CIRS-G = Cumulative Illness Rating Scale-Geriatric; GAD-7 = Generalized Anxiety Disorder 7; GDS = Geriatric Depression Scale; IADLs = instrumental activities of daily living; MoCA = Montreal Cognitive Assessment; OARS = Older Americans Resources and Services; *SD* = standard deviation.

^a^Higher is worse.

On average, participants had a wearing compliance rate of 0.87 (*SD* = 0.20) and had 6.7 prolonged inactive periods per day (*SD* = 1.98). We excluded outliers for prolonged inactivity periods (duration >3 *SD*s of the mean duration of total prolonged inactive periods per individual), which amounted to 2% of inactivity periods across participants.

### Dyadic Multilevel Modeling Results

Unconditional multilevel models revealed that, within couples, there was no significant difference between partners in the average number of inactive periods (*p* = .28). On average *across* couples, males and females had 6.90 (*SD* = 2.02) and 6.56 (*SD* = 1.93) inactive periods, respectively. There was a significant variability in the average number of physically inactive periods per day for both males and females.

Conditional models revealed that, for males, older age was the only variable associated with a greater number of inactive periods (β = 0.15, *p* = .002; Table 2). For females, more depressive symptoms in both dyad members were associated with fewer inactive periods (female: β = −0.30, *p* = .03; male: β = −0.41, *p* < .001). Additionally, for females, more dependency in completing their own IADLs predicted a greater number of inactive periods (β = 2.58, *p* < .001). Conditional models were adjusted for covariates. Finally, there was no evidence of a within-dyad interaction of men and women’s depressive symptoms on physically inactive periods in females (β = −0.03, *p* = .72).

**Table 2.** Multilevel Modeling Results (Adjusted)

Fixed effects		Unconditional						Conditional					
Gender	Variables	β	*SE*	*t*	*p* Value	Variance component	χ ^2^	β	*SE*	*t*	*p* Value	Variance component	χ ^2^
Male	Intercept	6.89	0.29	23.43	<.001			6.90	0.26	26.58	<.001		
	Male age							0.15	0.05	3.34	.002		
	Male depression							0.08	0.12	0.66	.51		
	Female depression							−0.03	0.15	−0.19	.85		
	Male IADL							−0.50	0.27	−1.86	.07		
	Female IADL							0.61	0.54	1.14	.26		
Female	Intercept	6.51	0.29	22.54	<.001			6.69	0.22	30.67	<.001		
	Male age							0.003	0.04	0.07	.95		
	Male depression							−0.41	0.10	−4.12	<.001		
	Female depression							−0.30	0.14	−2.19	.03		
	Male IADL							0.14	0.22	0.63	.53		
	Female IADL							2.58	0.70	3.69	<.001		
Male	Random effects					3.49	368.09					2.61	289.78
Female	Random effects					3.25	347.84					1.58	191.44

*Notes*: IADLs = instrumental activities of daily living; *SE* = standard error.

## Discussion and Implications

In this study of older adult couples, most of whom had at least one chronic condition, we examined the amount and determinants of physical inactivity by using a dyadic analysis and continuous actigraphy data. Although the amount of physical inactivity within couples was similar for males and females, across couples, there was significant variability in the amount of physical inactivity for both males and females. More physically inactive periods were associated with older age in male partners, whereas more inactive periods in females were associated with higher dependency in females’ IADLs. Additionally, more depressive symptoms in both dyad members were associated with less inactive periods in females. Considering the impact that mental health issues and decreased independence can have on the couple’s lifestyle, overall these results suggest that viewing the couple as a unit, rather than as separate individuals, may inform new potential dyadic pathways to reduce physical inactivity in older adults.

Findings in this study were gender-specific across couples. Greater dependency in IADLs was associated with more physical inactivity in females in this study, but not in males. Plausibly, IADLs provide an opportunity for females to avoid physical inactivity in their day-to-day lives (e.g., going shopping and doing housework) (Sheehan & Tucker-Drob, 2019). Thus, when females lose the capacity to complete IADL independently, an increase in their physical inactivity becomes evident. The lack of a similar association between males’ IADLs and physical inactivity may reflect a gendered division of household activities measured by the IADL scale, such as cooking, cleaning, and shopping (Sheehan & Tucker-Drob, 2019). These IADLs would not contribute as much to men’s physical inactivity when the functional ability is lost.

A second gender-specific finding is the effect of age on the number of inactive periods was observed in males but not females in this study. This finding is in line with a systematic review showing that sedentariness was higher as age increased, and males had higher sedentariness than females (Harvey et al., 2015). Worth mentioning, some of our couples were from rural communities (32 couples, 66.7%). There are health-related issues associated with rurality, including increased risk of morbidity and mortality, due to lifestyle differences and access to medical care (Lutfiyya et al., 2012). Thus, there may be additional environmental determinants associated with physical inactivity (e.g., community accessibility, residence layouts) that could disproportionately affect males as they age.

A third gender-specific finding in this study is the relationship between depressive symptoms and physical inactivity in females, but not in males. The discrepancy highlights the interdependent self-representation that women tend to exhibit more than men (Berg & Upchurch, 2007). It may be that as a result women attend more to the nurturing or caregiving role when they perceive distress in their partner (the caring/helping coping strategy), which would also involve more physical activity from IADLs as discussed above (Sharma et al., 2016). Alternatively, females may go out more to seek out social support to cope with either their own or males’ distress (the active engagement coping strategy; Pinquart & Sörensen, 2006).

Mental health issues are known to influence an individual’s activity level (Schuch et al., 2017). Although many studies suggest a positive correlation between physical inactivity and severe depressive symptoms, in the current study, physical inactivity in women was negatively associated with greater depressive symptoms in both members of the dyad (i.e., both actor and cross-partner effects). Interestingly, older women reported that breaking physical inactivity is a way to fight their own depression and boredom in a qualitative study (Chastin et al., 2014). Some expressed that standing up after a long sitting period is a coping mechanism against low mood. This reveals various behavior change/coping mechanisms are used in older women when they themselves or older men are depressed, not necessarily always becoming more sedentary.

When exploring the potential contagion of depression within couples and its impact on physical inactivity, there was no apparent within-dyad interaction. The insignificant result indicates that being in a couple where both dyad members are depressed did not explain additional variability in females’ inactivity. Instead, it is likely that women in this study exhibit more of a shared illness representation, and appraise depressive symptoms in either themselves or their partners as a stressor in the relationship (Berg & Upchurch, 2007), responding with less physical inactivity, potentially due to the reasons outlined above. Cautiously, this finding applies to a sample where both dyads are relatively healthy among the aging population, yet the men in the sample are older, sicker, and have more chronic conditions than the women.

Individuals generally perceive their partners to have the most influence over their physical activities compared to other health practices, such as medical treatment and sleep (Markey et al., 2007). Additionally, growing research suggests several avenues for collaborating with a partner on everyday health programs (Margrett & Willis, 2006; Rollo et al., 2016). A physical activity plan set by couples was more likely to be enacted than plans set by individuals (Keller et al., 2017). Partners’ involvement in exercise programs was also more cost-effective than individualized treatments (Lowery et al., 2014). Although dyadic interventions show promise, a meta-analysis conducted by Carr et al. (2019) showed that dyadic interventions yielded a small reduction in inactive time (Hedges’s *g* = 0.2). Most dyadic interventions provided cognitive-behavioral interventions, education, and family counseling. The small decrease in physical inactivity may be supported by our findings and previous studies. Our study found that an inactive lifestyle could, in part, be attributed to mental health issues and dependency in IADLs, for women, and for men it may be better explained by age-related changes. Similar results were reported where one member with physical disability could lead to increased physical inactivity for both of the married couple (Monin et al., 2016). Based on this evidence, we suggest that future studies may consider characteristics of the older adult couple, including age, weight, physical capacity, mental health, and decreased IADL independence in older adult couples as potential pathways to reduce inactivity.

We recognize that this study had certain limitations. Inactive periods may not be fully representative of an inactive lifestyle for several reasons. The data derived from wearable devices may indicate standing while not moving; however, this is less likely because it would require standing still for at least 30 min. The inactive periods may indicate participants just removed their watch for a period of time. We excluded outliers of prolonged inactive periods to account for this situation. An inactive period may vary from engagement in sedentary entertainment (e.g., watching television) to a socially stimulating conversation with a group of friends. Some sedentary activities such as chess or games may be less detrimental to health than other sedentary activities, such as television, due to the difference in cognitive demand that may translate to physiologic benefit. The nature of events is of particular importance because this may inform the strategies used to reduce inactive lifestyles. Last, there were possible motivational factors (e.g., lack of motivation, self-efficacy) that we did not include in the analysis (Rollo et al., 2016).

Our study has several strengths. We examined the inactivity of older adult couples, which is a different concept from exercise and physical activity, and much less commonly studied, but also an important contributing factor to achieve a healthy lifestyle. Previous studies that have monitored physical activity and/or inactivity through actigraphy or other self-reported assessments conducted over a short period of time (Gibbs et al., 2015). Studies that adopt a protocol of monitoring physical activity for only 7–14 days using wearable devices may not represent older adults’ typical behaviors due to initial elevation bias (Shrout et al., 2018). In other words, participants are likely to exhibit more physical activity and less physical inactivity than usual during the initial monitoring period when they are more aware of being observed. We used a month-long period of data, excluding the first month to avoid initial elevation bias, to consider average levels of physical inactivity from couples living in the same household. The unique data set derived from the CART initiative likely reflects real-world physical inactivity levels of older adults, more so than the 1–2-week protocols used in other studies. Further, dyadic analysis enabled us to address the interdependence and cross-partner phenomena of physical inactivity in older couples.

Reducing inactivity is a growing interest in the aging population when exercise becomes a challenging option. Viewing couples’ activity as a unit is a promising approach to yield lifestyle changes and promote healthy practices. The consideration of demographics, mental health issues, and IADL dependence of dyads can be built upon current interventions to develop tailored interventions for older adults.

## Supplementary Material

igaa066_suppl_Supplementary_Table_1
