# Corrigendum to: Prolonged Physical Inactivity in Older Adult Couples: A Dyadic Analysis Using Actigraphy

**DOI:** 10.1093/geroni/igab036

**Published:** 2021-11-08

**Authors:** Chao-Yi Wu, Lyndsey M Miller, Rachel N Wall, Zachary T Beattie, Lisa C Silbert, Jeffrey A Kaye

**Affiliations:** 1 Department of Neurology, Oregon Health & Science University, Portland, USA; 2 Oregon Center for Aging and Technology (ORCATECH), Oregon Health & Science University, Portland, USA; 3 School of Nursing, Oregon Health & Science University, Portland, USA; 4 Department of Neurology, Veterans Affairs Portland Health Care System, Portland, Oregon, USA

In the original research article, “Prolonged Physical Inactivity in Older Adult Couples: A Dyadic Analysis Using Actigraphy,” DOI: 10.1093/geroni/igaa066, under the Funding section, “…the Collaborative Aging-in-place Research Using Technology (CART) initiative (National Institutes of Health [grant number U2C AG0543701 to J. A. Kaye]” has been replaced by “…the Collaborative Aging-in-place Research Using Technology (CART) initiative (National Institutes of Health [grant number U2C AG054397 to J. A. Kaye].

