# Supplementary material for: Prolonged Physical Inactivity in Older Adult Couples: A Dyadic Analysis Using Actigraphy
Source: Innov Aging. 2020 Dec 30;5(1):igaa066. doi: 10.1093/geroni/igaa066 (PMC7937911; doi:10.1093/geroni/igaa066)
Supplement: igaa066_suppl_Supplementary_Table_1 [file igaa066_suppl_Supplementary_Table_1.docx]

**Supplementary Table 1**. Zero-Order Correlation Matrix

|  | Variables | 1 | 2 | 3 | 4 | 5 | 6 | 7 | 8 | 9 | 10 | 11 | 12 | 13 | 14 | 15 | 16 |
| --- | --- | --- | --- | --- | --- | --- | --- | --- | --- | --- | --- | --- | --- | --- | --- | --- | --- |
| 1 | Inactivity periods (M) ^a^ | - |  |  |  |  |  |  |  |  |  |  |  |  |  |  |  |
| 2 | Inactivity periods (F) | .02 | - |  |  |  |  |  |  |  |  |  |  |  |  |  |  |
| 3 | Age (M) | .34* | -.01 | - |  |  |  |  |  |  |  |  |  |  |  |  |  |
| 4 | Age (F) | .28 | .09 | .76* | - |  |  |  |  |  |  |  |  |  |  |  |  |
| 5 | Cognition (M) | .02 | .15 | -.09 | .02 | - |  |  |  |  |  |  |  |  |  |  |  |
| 6 | Cognition (F) | -.20 | -.08 | .03 | -.06 | -.01 | - |  |  |  |  |  |  |  |  |  |  |
| 7 | Comorbidity (M) | .03 | -.30* | .25 | .05 | -.12* | .22 | - |  |  |  |  |  |  |  |  |  |
| 8 | Comorbidity (F) | .18 | .17 | .22 | .39* | .03 | -.30* | .24 | - |  |  |  |  |  |  |  |  |
| 9 | Depressive symptoms (M) | -.09 | -.29 | -.03 | -.34* | -.01 | .17 | .47* | -.22 | - |  |  |  |  |  |  |  |
| 10 | Depressive symptoms (F) | .22 | .04 | .05 | .03 | .11 | -.19 | .15 | .30* | .05 | - |  |  |  |  |  |  |
| 11 | Anxiety (M) | -.10 | -.27 | -.32* | -.45* | -.13 | .22 | .27 | -.20 | .73* | -.07 | - |  |  |  |  |  |
| 12 | Anxiety (F) | .24 | -.11 | -.05 | -.23 | -.04 | .03 | .31* | .04 | .42* | .65* | .30* | - |  |  |  |  |
| 13 | IADL (M) | .09 | -.08 | .42* | .16 | -.32* | .17 | .43* | -.08 | .19 | -.04 | .004 | -.07 | - |  |  |  |
| 14 | IADL (F) | .10 | .31* | .02 | .08 | .25 | -.33* | .01 | .32* | -.17 | .43* | -.19 | .02 | -.04 | - |  |  |
| 15 | Gait speed (M) | .07 | -.22 | .36* | .14 | -.28 | .02 | .48* | .02 | .27 | .07 | .21 | -.01 | .67* | -.01 | - |  |
| 16 | Gait speed (F) | .24 | .22 | .14 | .17 | .02 | -.17 | .31* | .44* | -.05 | .46* | -.14 | .31* | .07 | .24 | .005 | - |
| *Notes*: M=male; F=female; IADL=instrumental activities of living.  ^a^ Higher scores indicate more.  **p* <.05 | | | | | | | | | | | | | | | | | |
